# Essential list of medicinal products for rare diseases: recommendations from the IRDiRC Rare Disease Treatment Access Working Group

**DOI:** 10.1186/s13023-021-01923-0

**Published:** 2021-07-13

**Authors:** William A. Gahl, Durhane Wong-Rieger, Virginie Hivert, Rachel Yang, Galliano Zanello, Stephen Groft

**Affiliations:** 1 National Human Genome Research Institute, NIH, Bethesda, USA; 2 Canadian Organization for Rare Disorders, Toronto, Canada; 3 EURORDIS-Rare Diseases Europe, Paris, France; 4 China Alliance for Rare Diseases, Geneva, Switzerland; 5 IRDiRC Scientific Secretariat, Inserm, Paris, France; 6 National Center for Advancing Translational Sciences, NIH, Bethesda, USA

## Abstract

**Background:**

Treatments are often unavailable for rare disease patients, especially in low-and-middle-income countries. Reasons for this include lack of financial support for therapies and onerous regulatory requirements for approval of drugs. Other barriers include lack of reimbursement, administrative infrastructure, and knowledge about diagnosis and drug treatment options. The International Rare Diseases Research Consortium set up the Rare Disease Treatment Access Working Group with the first objective to develop an essential list of medicinal products for rare diseases.

**Results:**

The Working Group extracted 204 drugs for rare diseases in the FDA, EMA databases and/or China’s NMPA databases with approval and/or marketing authorization. The drugs were organized in seven disease categories: metabolic, neurologic, hematologic, anti-inflammatory, endocrine, pulmonary, and immunologic, plus a miscellaneous category.

**Conclusions:**

The proposed list of essential medicinal products for rare diseases is intended to initiate discussion and collaboration among patient advocacy groups, health care providers, industry and government agencies to enhance access to appropriate medicines for all rare disease patients throughout the world.

**Supplementary Information:**

The online version contains supplementary material available at 10.1186/s13023-021-01923-0.

## Introduction

A significant unmet need for individuals living with rare diseases is access to beneficial therapies, even those that are approved by major regulatory bodies and are considered as standards of care by experts throughout the world. This issue is especially apparent in low-and-middle-income countries (LMICs) [1] but also affects a substantial proportion of eligible patients in high-income jurisdictions. Of course, this inequity in access applies not only to rare disease drugs but also therapies for common, chronic diseases [2]. However, the disparity is even greater for rare disease treatments [3]. Moreover, while there are international initiatives and programs to make available therapies for conditions affecting large patient populations, such as diabetes, HIV and cancer, there has been little action to improve access to drugs for those suffering from rare conditions [4].

To stimulate a broad response to this unmet need, the International Rare Diseases Research Consortium (IRDiRC) established the Rare Disease Treatment Access Working Group (RDTAWG) with three aims: (1) To improve standards of care for RD patients by promoting access to approved medicines; (2) To initiate research into the barriers to accessing RD drugs, especially in LMICs; and (3) To define opportunities to address those barriers.

This paper is the first of a three-part series with special focus on lack of access to orphan and rare disease drugs in LMICs and also inequitable access in high-income countries. This first paper presents a curated list of medicines considered to be essential for rare disorders and already approved by the US Food and Drug Administration (FDA), the European Medicines Agency (EMA), and/or China’s National Medical Products Administration (NMPA). The second paper will discuss the barriers to access stratified by types of therapy, characteristics of rare disease populations, and key country parameters such as investment in health, health system capabilities, and rare disease priorities. That paper will also review some existing mechanisms for providing therapeutic access for rare and non-rare conditions. The third paper will consider strategies for improving access directed toward barriers identified along the patient pathway, in general and specific to rare conditions.

## Methods

The IRDiRC RDTAWG developed a list of essential medicinal products for rare conditions; the list was not intended to include all medicines used to treat rare diseases but those that could be considered as essential based on approvals by key regulatory agencies in the USA, the European Union (EU) and China for the treatment of rare conditions. Two approaches were used to compile the list. The first approach was to start with databases of medicinal products with designated orphan status or marketing authorizations for rare disease indications. The initial references were the USA FDA Orphan Drug Product Designation database for products approved in the USA [5], the Orphanet list of medicinal products for rare diseases in Europe (2020) [6], and the EMA database of approved products and designations [7]. All drugs with orphan designations and FDA approval were extracted and a list was created, arranged by rare condition usage, generic (medicinal) name, and regulatory approval status. Medicinal products for rare diseases that have European Union marketing authorizations (with or without orphan drug designation) were then collated by using the Orphanet and EMA databases. To round out the list, China’s first published Rare Diseases Catalog [8, 9] of 121 rare diseases/disease types and China’s Lists of Novel Drugs Approved in Other Jurisdictions with Urgent Clinical Needs [10–12] were consulted to develop a list of medicines that were approved for the treatment of recognized rare conditions.

The second approach to developing the essential rare disease medicines list was to start with the World Health Organization Model List of Essential Medicines—21st list, 2019 [13] and the WHO Model List of Essential Medicines for Children—7th list, 2019 [14] to extract all essential medicines that were indicated for the treatment of rare diseases. This exercise identified 26 medicines on the FDA, EMA, and/or China NMPA lists that were also on the WHO essential medicines lists; however, the WHO indication was often not for a rare disease but a more common condition. Some key exceptions are medicines for treating hemophilia, cystic fibrosis, Marfan syndrome, Prader–Willi syndrome, myasthenia gravis, and sickle cell disease.

It is important to note that this collated list does not include any rare cancer drugs. Given the large number and the uniqueness, rare cancers deserve a separate list [15]. The European Joint Action on Rare Cancers initiated this work by conducting a survey for health professionals leading to the identification of 68 essential medicines for the treatment of pediatric malignancies according to best standards [16].

The core WG collated the initial list of medicinal products by eliminating duplicates and combining medicines that were ostensibly versions of a single drug therapy. While strict inclusion and exclusion criteria were not employed in compiling the RD drug list, all the entries were required to be approved by a major regulatory agency. In addition, all were recognized by the authors and the consultant group to have a reasonable risk/benefit ratio and to represent accepted treatments for the diseases under consideration. Drugs were excluded if they had orphan designation but lacked approval, if approval was withdrawn, or if the drug was known to have unacceptable side effects. Some medications in Table 1 are indicated for common disorders but are also treatments for rare subsets of the disorder (e.g., pediatric ulcerative colitis).

**Table 1:** List of essential medicinal products for rare diseases

	Condition	Drug	Approvals	WHO
*Metabolic*				
	Aminoacid Disorders			
	Urea cycle disorders	Benzoate and phenylacetate	FDA	
		Sodium phenylbutyrate	FDA, EMA	
	N-acetylglutamate synthetase deficiency	Carglumic acid	FDA, EMA	
	Homocystinuria	Betaine	FDA, EMA	
	Hyperphenylalaninemia	Sapropterin	FDA, EMA, NMPA	
	Tetrahydrobiopterin deficiency	Sapropterin	FDA, EMA, NMPA	
	Phenylketonuria	Pegvaliase	FDA, EMA	
	Tyrosinemia type 1	Nitisinone	FDA, EMA	
	Alkaptonuria	Nitisinone	EMA	
	Lysosomal Storage Diseases			
	Gaucher disease	Miglustat	FDA, EMA, NMPA	
		Eliglustat	FDA, EMA	
		Velaglucerase alfa	FDA, EMA	
		Imiglucerase	FDA, EMA, NMPA	
		Taliglucerase	FDA, EMA	
	Fabry disease (alphagalactosidase A deficiency)	Agalsidase beta	EMA, NMPA	
		Agalsidase alfa	EMA, NMPA	
		Migalastat	FDA, EMA	
	Lysosomal acid lipase deficiency, Wolman disease, Cholesteryl ester storage disease	Sebelipase alfa	FDA, EMA	
	Pompe disease	Alglucosidase alfa	FDA, EMA, NMPA	
	Alpha mannosidosis	Velmanase alfa	EMA	
	Mucopolysaccharidosis I (Iduronidase deficiency)	Laronidase	EMA, NMPA	
	Hunter syndrome (Mucopolysaccharidosis II)	Idursulfase	FDA, EMA, NMPA	
	Mucopolysaccharidosis IV (Morquio A syndrome)	Elosulfase alfa	FDA, EMA, NMPA	
	Mucopolysaccharidosis VI (Maroteaux-Lamy syndrome)	Galsulfase	EMA	
	Mucopolysaccharidosis VII (Sly syndrome)	Vestronidase alfa	FDA, EMA	
	Neuronal ceroid lipofuscinosis type 2	Cerliponase alfa	FDA, EMA	
	Nephropathic cystinosis	Cysteamine	FDA, EMA	
		Cysteamine (enteric coated)	FDA, EMA	
		Cysteamine hydrochloride eyedrops	FDA, EMA	
	Cholesterol, Lipid, Fatty Acid Disorders			
	Homozygous familial hypercholesterolemia	Evolocumab	FDA, EMA, NMPA	
		Rosuvastatin calcium	FDA, NMPA	
		Lomitapide	FDA, EMA	
	Cholesterol and bile acid synthesis defects	Cholic acid	FDA, EMA, NMPA	
	Cerebrotendinous xanthomatosis	Chenodeoxycholic acid	EMA	
	Familial chylomicronemia syndrome	Volanesorsen	EMA	
	Congenital or hereditary chronic cholestasis	Tocofersolan	EMA	
	Other Metabolic Disorders			
	Pediatric onset hypophosphatasia	Asfotase alfa	FDA, EMA	
	Hypophosphatemic rickets (x-Linked)	Burosumab-twza	FDA, EMA	
	Hyperphosphatemia in renal failure	Calcium acetate	FDA	
	Osteogenesis imperfecta	Alendronate	NMPA	
	Scurvy	Ascorbic acid	FDA	WHO
	Metabolic acidosis	Thiamine	EMA	WHO
		Trisodium citrate	EMA	
	Genetic carnitine deficiency	Levocarnitine	FDA, NMPA	
	Fatty acid oxidation disorders	Triheptanoin	FDA	
	Acyl Coenzyme A dehydrogenase deficiency	Riboflavin	EMA	WHO
	Hereditary orotic aciduria	Uridine triacetate	FDA	
	Prevention of uric acid nephrolithiasis	Potassium citrate	FDA	
	Prevention of cystine nephrolithiasis (cystinuria)	Tiopronin	FDA	
	Wilson disease	Penicillamine	NMPA	WHO
		Trientine HCl	FDA, EMA	
		Zinc acetate	FDA, EMA	
	Cobalamin defects	Hydroxocobalamin	FDA	WHO
*Neurologic*				
	General			
	Transthyretin amyloidosis	Inotersen	FDA, EMA	
		Tafamidis	FDA, EMA	
		Patisiran sodium	FDA, EMA	
	Multiple Sclerosis	Teriflunomide	EMA, NMPA	
		Fingolimod HCl	EMA, NMPA	
		Siponimod	NMPA	
	Parkinson Disease (Young and Early-onset)	Rasagiline	EMA, NMPA	
		Selegiline	FDA, EMA, NMPA	
		Pramipexole	EMA, NMPA	
		Carbidopa/Levodopa	FDA, EMA	WHO
	Narcolepsy with cataplexy	Pitolisant	FDA, EMA	
		Sodium oxybate	FDA, EMA	
	Huntington Disease	Deutetrabenazine	NMPA	
		Tetrabenazine	FDA, EMA	
	Dystonia, Spasticity	Baclofen	FDA	
	Tuberous Sclerosis Complex	Everolimus	FDA, EMA	
	Spina bifida (prevention)	Folic acid	EMA	WHO
	Biotinidase deficiency	Biotin	NMPA	
	Epilepsy			
	Infantile spasms	Vigabatrin	FDA, EMA	
	Lennox-Gastaut syndrome	Rufinamide	FDA, EMA	
		Cannabidiol	FDA, EMA	
	Severe myoclonic epilepsy in infancy (Dravet syndrome)	Stiripentol	FDA, EMA	
	Status epilepticus	Midazolam	FDA	WHO
	Juvenile myoclonic epilepsy, Generalized epilepsy	Levetiracetam	EMA	
	Complex and rare disease epilepsy	Clobazam	FDA	
		Lamotrigine	FDA	WHO
		Topiramate	FDA	
	Neuromuscular Diseases			
	Amyotrophic lateral sclerosis	Gabapentin	FDA	
		Riluzole	FDA, EMA, NMPA	
		Radicava	NMPA	
	Myasthenia gravis	Pyridostigmine Bromide	NMPA	WHO
	Lambert-Eaton myasthenic syndrome	Amifampridine	EMA	
	Non-dystrophic myotonic disorders	Mexiletine hcl	EMA	
	5q Spinal Muscular Atrophy	Nusinersen sodium	FDA, EMA, NMPA	
*Hematologic*				
	Coagulation Defects			
	Hemophilia A (Factor VIII deficiency)	Octocog alpha	EMA	
		Rurioctocog alfa pegol	EMA	
		Lonoctocog alfa	EMA	
		Emicizumab	FDA, EMA, NMPA	
		Damoctocog alfa pegol	EMA	
		Turoctocog alpha	EMA	
		Simoctocog alfa	EMA	
		Moroctocog alpha	EMA	
		Desmopressin acetate	FDA, EMA	WHO
		Recombinant Factor VIII	EMA, NMPA	
		Efmoroctocog alfa	EMA	
	von Willebrand disease	Factor VIII/ von Willebrand factor	EMA	
		Vonicog alfa	EMA	
	Hemophilia B (Factor IX deficiency)	Eftrenonacog alfa	EMA	
		Albutrepenonacog alfa	EMA	
		Nonacog alpha	EMA	
		Human coagulation factor IX	EMA	WHO
		Nonacog beta pegol	EMA	
		Nonacog gamma	EMA	
		Recombinant Factor IX	EMA, NMPA	
	Hemophilia (Factor VII deficiency)	Eptacog alpha (activated)	EMA	
		Recombinant Factor VIIa	EMA	
	Factor X deficiency	Human coagulation factor X	EMA	
	Factor XIII A-subunit deficiency	Catridecacog	EMA	
	Protein C deficiency	Human protein c	EMA	
	Anemias			
	Sickle cell anemia	Hydroxyurea	FDA	
	Anemia of end-stage renal disease	Epoetin alfa	FDA	WHO
	Idiopathic thrombocytopenic purpura, Aplastic anemia	Eltrombopag	FDA, EMA	
	Beta thalassemia major	Deferasirox	FDA, EMA, NMPA	
	Other Hematologic Disorders			
	Congenital and acquired methemoglobinemia	Methylene blue injection	FDA	
	Acute intermittent porphyria	Hemin	FDA	
	Erythropoietic protoporphyria	Afamelanotide	FDA, EMA	
	Multicentric Castleman’s disease	Siltuximab	FDA, EMA	
	Essential thrombocythemia	Anagrelide hydrochloride	FDA, EMA	
	Paroxysmal nocturnal hemoglobinuria	Ravulizumab	FDA, EMA	
	Severe congenital neutropenia	Macapegfilgrastim	NMPA	
	Conditioning for hematopoietic stem cell transplant	Busulfan	FDA, EMA	
		Thiotepa	FDA, EMA	
	Iron overload	Deferiprone	FDA, EMA	
	Acquired thrombotic thrombocytopenic purpura	Caplacizumab	FDA, EMA	
	Immune (idiopathic) thrombocytopenic purpura	Romiplostim	FDA, EMA	
	Polycythemia vera	Ropeginterferon alfa-2b	EMA	
		Ruxolitinib	FDA, EMA	
	Agammaglobulinemia	Immunoglobulin infusion	NMPA	
*Inflammatory*				
	Rheumatoid Arthritis			
	Juvenile rheumatoid arthritis	Methotrexate	FDA, EMA	WHO
		Etanercept	FDA, EMA	
		Methylprednisolone	EMA	WHO
		Adalimumab	FDA	
		Infliximab	FDA	
		Tocilizumab	FDA, EMA	
		Abatacept	EMA	
		Golimumab	FDA, EMA	
	Gastrointestinal Inflammation			
	Pediatric Crohn's disease	Adalimumab	FDA	
		Infliximab	FDA	
	Pediatric ulcerative colitis	Mesalamine	FDA	
		5-aminosalicylic acid	FDA	
		Adalimumab	FDA	
		Infliximab	FDA	
	Primary biliary cholangitis	Obeticholic acid	FDA, EMA	
	Hereditary chronic cholestasis	Tocofersolan	EMA	
	Angioedema			
	Hereditary angioedema	C1 inhibitor(human)	EMA	
		Icatibant acetate	FDA, EMA	
		Lanadelumab	FDA, EMA, NMPA	
		Danazol	NMPA	
		Tranexamic acid	FDA, NMPA	WHO
	Angioedema due to C1 esterase inhibitor deficiency	C1-esterase-inhibitor, human	FDA	
		Conestat alfa	EMA	
	Other Inflammatory Disorders			
	Multiple sclerosis, Behcet's disease, Familial Mediterranean fever	Colchicine	FDA, NMPA	
	Dermatomyositis, Atypical hemolytic uremic syndrome, Neuromyelitis Optica, Paroxysmal nocturnal hemoglobinuria, Myasthenia gravis	Eculizumab	FDA, EMA, NMPA	
	Anti-neutrophil vasculitis, Wegener’s granulomatosis, Churg-Strauss Syndrome	Rituximab	FDA	WHO
	Familial Mediterranean fever, Cryopyrin fevers	Canakinumab	FDA, EMA, NMPA	
	Still's disease, Systemic juvenile arthritis	IL-1 Receptor antagonist anakinra	FDA, EMA	
	Neurotrophic keratitis	Cenegermin	FDA, EMA	
	Vernal keratoconjunctivitis	Ciclosporin	EMA	WHO
	Non-infectious uveitis	Dexamethasone	FDA, EMA	WHO
	Cryopyrin-associated periodic syndromes	Rilonacept	FDA, EMA	
*Endocrine*				
	Growth hormone deficiency in children	Somatropin for injection	FDA, EMA	
	Acromegaly	Octreotide	FDA	
		Lanreotide	FDA	
		Pegvisomant	FDA, EMA	
		Pasireotide	FDA, EMA	
	Endogenous Cushing’s syndrome	Osilodrostat	FDA, EMA	
		Ketoconazole	EMA	
	Adrenal insufficiency	Hydrocortisone	FDA, EMA, NMPA	WHO
	Idiopathic Hypogonadotropic Hypogonadism	Human chorionic gonadotropin	EMA, NMPA	
		Gonadotropin-releasing hormone	EMA, NMPA	
	Primary insulin-like growth factor-1 deficiency	Mecasermin	FDA, EMA	
	Paget's disease (osteitis deformans)	Calcitonin-human for injection	FDA	
	Hypoparathyroidism	Parathyroid hormone	FDA, EMA	
	Non-24-h sleep–wake disorder	Tasimelteon	FDA, EMA	
	Leptin deficiency in lipodystrophy patients	Metreleptin	FDA, EMA	
	Familial partial lipodystrophy	Metreleptin	EMA	
*Pulmonary*				
	Pulmonary arterial hypertension	Macitentan	FDA, EMA, NMPA	
		Tadalafil	FDA, EMA	
		Ambrisentan	FDA, EMA, NMPA	
		Nitric oxide	FDA, EMA	
		Sildenafil	EMA, NMPA	
		Bosentan monohydrate	FDA, EMA, NMPA	
		Selexipag	FDA, EMA, NMPA	
		Iloprost	FDA, EMA, NMPA	
		Parenteral treprostinil	FDA, EMA, NMPA	
		Riociguat	FDA, EMA, NMPA	
	Cystic fibrosis	Mannitol	FDA, EMA	WHO
		Ivacaftor	FDA, EMA	
		Tezacaftor/ivacaftor	FDA, EMA	
		Tobramycin	FDA, EMA	
		Aztreonam	FDA, EMA	
		Colistimethate sodium	EMA	
		Lumacaftor / ivacaftor	FDA, EMA	
		Levofloxacin	EMA	WHO
	Idiopathic Pulmonary Fibrosis	Pirfenidone	FDA, EMA	
		Nintedanib	FDA, EMA, NMPA	
	Primary apnea of premature newborns	Caffeine citrate	FDA, EMA	WHO
	Lymphangioleiomyomatosis, Tuberous sclerosis	Sirolimus	FDA, EMA	
*Immunologic*				
	Severe combined immunodeficiency, Adenosine deaminase deficiency	Pegademase bovine	FDA	
		CD34 + cells transduced with ADA cDNA	EMA	
	Chronic granulomatous disease	Interferon gamma 1-b	FDA	
*Miscellaneous*				
	Mastocytosis	Cromolyn sodium	FDA	
	Ventricular tachycardia	Amiodarone	FDA	WHO
	Limbal stem cell deficiency	Autologous human corneal stem cells	EMA	
	Inherited retinal dystrophy	Voretigene neparvovec	FDA, EMA	
	Short bowel syndrome	Teduglutide	FDA, EMA	
	Hepatic veno-occlusive disease, Sinusoidal obstruction	Defibrotide	FDA, EMA	
	Autosomal dominant polycystic kidney disease	Tolvaptan	FDA, EMA	
	Patent ductus arteriosus	Ibuprofen	FDA, EMA	WHO
	Anthracycline extravasation	Dexrazoxane	FDA, EMA	

List of 204 essential medicinal products for rare diseases with marketing authorization extracted from the FDA database and/or EMA database and/or Chinas’s Rare Diseases Catalog

The goal of the RDTAWG for this first stage of work was identified as the creation of a list of RD medicines that, based on orphan designation and approval or marketing authorization, were efficacious, safe and having a significant impact on the quality and/or duration of life. In some cases, they could be considered standards of care based on widespread and long-term use; however, no attempt was made to categorize the drugs according to life-saving, curative, or beneficial properties.

Moreover, while it was desirable that the medicines on the list could be managed across a variety of countries at different stages of health system development, there was no detailed assessment on the basis of cost-effectiveness, complexity of management, or requirements for administration. Hence, unlike the WHO list of essential medicines, this list of RD drugs is not stratified nor prioritized on the basis of various criteria that could affect feasibility of adoption.

This list is intended to be the initial iteration of a “living document”, to be revised and updated periodically. The list is not based on definitive criteria for inclusion nor is it the product of an expert consensus process. It is not intended to be comprehensive but is proposed to the rare disease community for consideration and uptake as well as a starting point or guide for jurisdictions to set policies on provision of rare disease medicines to their populations.

The compiled list was sent to a group of rare disease specialists (as listed in Acknowledgments) with instructions to review those medications within their area(s) of expertise and, specifically, to eliminate duplicate or redundant medicines, remove drugs considered inappropriate or ineffective, add other drugs that should be on the list, and provide comments as appropriate. Feedback was discussed by all members of the core RDTAWG to arrive at a consensus whether to accept recommendations, or not, to arrive at a final collated list.

## Results

The Table 1 presents the current working list of essential rare disease medicines with different versions of a medication listed separately where appropriate. The list is organized into seven disease categories: metabolic, neurologic, hematologic, anti-inflammatory, endocrine, pulmonary, and immunologic, plus a miscellaneous category. Moreover, some diseases cluster together in groups (e.g. Wolman disease and Cholesteryl ester storage disease cluster in the group of Lysosomal acid lipase deficiency). However, in identifying the total number of diseases, these diseases are counted individually and not as a single cluster. Within each category, drugs are listed by subgroupings and specific conditions, with multiple indications where appropriate. The third column gives the agencies that approved the drug; the fourth column notes drugs that are on the WHO Model List of Essential Medicines (21st edition). Some of the drugs have indications beyond those listed in the table. The drugs are not coded in terms of priority, therapeutic strength or equivalence, need for specialized diagnosis or care, or any restrictions (cf. WHO Model List of Essential Medicines). The greatest number of drugs is in the metabolic disease category, but various neurological diseases are extensively represented.

Table 2 provides a summary on the total number of listed diseases, the number of diseases per category, the number of total medicinal products, the number of drugs and biologics, the number of medicinal products per disease category, the number of medicinal products treating more than one disease, and the number of medicinal products approved in each jurisdiction. A total of 134 diseases are listed; among these, the largest category consists of metabolic conditions (n = 40), followed by hematologic conditions (n = 24), inflammatory (n = 23), neurologic (n = 20), endocrine (n = 11), miscellaneous (n = 9), pulmonary (n = 5) and immunologic (n = 2).

**Table 2:** Number of diseases and medicinal products per category

	Number
*Diseases*	
Total	134
Metabolic/inborn errors	40
Neurologic	20
Hematologic	24
Inflammatory	23
Endocrine	11
Pulmonary	5
Immunologic	2
Miscellaneous	9
*Medicinal products*	
Total medicinal products (MP)	204
Drugs	125
Biologics	79
MP for metobolic diseases	51
MP for neurologic diseases	34
MP for hematologic diseases	43
MP for inflammatory diseases	28
MP for endocrine diseases	15
MP for pulmonary diseases	22
MP for immunologic diseases	3
MP for miscellaneous	9
MP used to treat multiple diseases	6
MP approved by the FDA	139
MP approved by the EMA	160
MP approved by the NMPA	51
MP on the WHO list of essential medicines (2019)	25

Summary of the total number of listed diseases, the number of diseases per category, the number of total medicinal products, the number of drugs and biologics, the number of medicinal products per disease category, the number of medicinal products treating multiple diseases, and the number of medicinal products approved in each jurisdiction

Additionally, as noted in Table 2, the list includes 204 drugs, of which 125 are chemical drugs and 79 are classified as biologics, which also includes recombinant proteins, polyclonal and monoclonal antibodies, and cell and nucleic acid therapies. There are six drugs that treat more than one disease (Additional file 1).

## Discussion

Individuals with rare diseases encounter many challenges along the path to appropriate care and treatment. The first obstacle for many is obtaining an accurate diagnosis, which often takes more than 5 years [17]. For many, the next hurdle involves finding expert care and treatment, which can vary depending upon many factors including geographic location and socioeconomic status. In fact, researchers have noted profound disparities across the globe in access to rare disease medicines, with significant impact on health outcomes and quality of life [18, 19]. In 2006, Stolk et al. [20] called for inclusion of RD drugs as essential medicines, but this has not occurred.

Many of the drugs in our RD drug list are not included in the WHO Model List of Essential Medicines.

We note that not all of the drugs on our list are approved across all jurisdictions, and a few with regulatory approval and/or marketing authorization are not indicated for the specified rare disease(s), even if they are recognized as a standard of care or appropriate. Based upon such a lack of indication, some health systems may choose to deny reimbursement even if the drug is inexpensive, genericized and in distribution. This problem affects patients in high-income as well as low-and-middle income countries. Therefore, it is important to take a broader contextual approach to understand the challenges rare disease patients are facing and address them collectively and systematically. Moreover, some of the drugs listed in Table 1 are applicable to only a fraction of the patients who have the associated disease. Ivacaftor for certain genetic subtypes of cystic fibrosis is one example.

Approximately one-third of all persons worldwide, including those in low-income but also middle-income countries, do not have access to essential medicines, specifically drugs, vaccines, and diagnostics for communicable, noncommunicable, social-behavioral illnesses, and emerging environmentally induced diseases [1]. The cause of the problem, like the cause of the diseases, is multifactorial and requires not only multidisciplinary and multisectoral approaches but integrated, holistic innovative solutions. Barriers at the individual level include the lack of health literacy, awareness of therapies, and advocacy capacity. Healthcare professionals similarly may lack awareness of appropriate medicines, knowledge to use effectively, and capacity to advocate for access. Major impediments at the systems level include lack of lower cost alternatives (generics and biosimilars) as well as the lack of regulatory, clinical and infrastructure capacity to make complex innovative therapies available and to deliver them to patients [21]. For example, depending of the type of therapies (chemical drug/biologic/gene or cell therapy), countries or jurisdictions may have to consider the feasibility of establishing and maintaining manufacturing and delivery chains relative to their local capacities. In addition, while nations may be criticized for limited national commitment to healthcare and insufficient investment in universal health coverage, criticism may also be levied on industry for the lack of transparency and high pricing that compromise a nation’s ability to deliver optimal healthcare; this critique is punctuated by the WHO resolution on disclosure of drug prices [22].

Many of the aforementioned challenges (especially regulatory expertise and clinical capacity) have a disproportionate impact on rare disease drugs and patients, but there are additional barriers. Some are grounded in “high evidential uncertainty” in extending clinical trial data to real-world outcomes. This is highly problematic in countries that apply “traditional” health technology assessment (HTA) or value-based assessment (VBA) methodology to RD therapies compared to those jurisdictions that use supplemental processes with greater flexibilities that treat RD treatments differently [23].

How could this list of RD medicines be used? A potential pathway is one based on EMA’s EU-Medicines4all (EUM4all) procedure. EMA established EUM4all to provide expert reviews on benefits and risks of medicines that would be used outside the EU, with emphasis on LMICs [24]. Subsequent analysis found that 138 regulatory approvals had been granted in 90 different countries worldwide for six medicines based on EUM4all opinions, with acknowledged great public health impact.

The EUM4all initiative dealt with a broad range of medicines with high impact in LMICs, but we propose that the procedure could profitably be applied to RD medicines. This paper is intended to elicit suggestions and call for collaborations on how to modify, disseminate, and use the list of medicines in the Table 1. Specifically, the RDTAWG seeks input from RD advocacy groups, healthcare providers, pharmaceutical companies, and government agencies. Subsequent actions include a conference to bring together key stakeholders to elaborate on the list, identify barriers and opportunities for application and collaborate on next steps. The ultimate goal is to enhance access to appropriate medicines for all rare disease patients throughout the world.

## Conclusions

The limited number of approved therapeutic options, combined with the unavailability of existing treatments, significantly impair the life of rare disease patients in LMICs. While many countries have recently developed policies and regulations for rare diseases and orphan drugs, access to treatment remains variable among LMICs. With the vision of leaving no one behind, the IRDiRC RDTAWG used the FDA, EMA and China NMPA databases to extract approved drugs and create the first list of 204 essential medicinal products for rare diseases. The list was organized into seven disease categories, excluding rare cancers and rare infectious diseases. The ultimate goal of this list is to further stimulate interactions among patient organizations, health care providers, industry and government agencies to improve standards of care for rare diseases by promoting access to treatments.

## Supplementary Information

**Additional file 1**. Used to complement the information provided in Table 2. Individual diseases (and not cluster of diseases) are listed by alphabetic order for each category. Medicinal products are filtered by alphabetic order and listed for each disease category. The 6 medicinal products used to treat multiple diseases and the 79 biologics are listed.

## Data Availability

The datasets analyzed as sources for the lists are available from the corresponding author upon request.

## Abbreviations

IRDiRC: International Rare Diseases Research Consortium
RDTAWG: Rare Disease Treatment Access Working Group
RD: Rare Diseases
LMICs: Low-and-Middle-Income Countries
OMP: Orphan Medicinal Products
FDA: US Food and Drug Administration
EMA: European Medicines Agency
WHO: World Health Organization
EU: European Union
NMPA: National Medical Products Administration of China
HTA: Health Technology Assessment
VBA: Value Based Assessment
